# Crystal structure of the S187F variant of human liver alanine: Aminotransferase associated with primary hyperoxaluria type I and its functional implications

**DOI:** 10.1002/prot.24300

**Published:** 2013-06-01

**Authors:** Elisa Oppici, Krisztian Fodor, Alessandro Paiardini, Chris Williams, Carla Borri Voltattorni, Matthias Wilmanns, Barbara Cellini

**Affiliations:** 1 Department of Life Sciences and Reproduction, Section of Biological Chemistry, University of VeronaStrada Le Grazie, 8 37134, Verona, Italy; 2 EMBL c/o DESYNotkestrasse 85, Hamburg, Germany; 3 Department of Biochemical Sciences “A. Rossi Fanelli”, University “La Sapienza”00185, Roma, Italy

**Keywords:** alanine:glyoxylate aminotransferase, Primary Hyperoxaluria Type I, pyridoxal 5′-phosphate, crystal structure, pathogenic variant, molecular modeling

## Abstract

The substitution of Ser187, a residue located far from the active site of human liver peroxisomal alanine:glyoxylate aminotransferase (AGT), by Phe gives rise to a variant associated with primary hyperoxaluria type I. Unexpectedly, previous studies revealed that the recombinant form of S187F exhibits a remarkable loss of catalytic activity, an increased pyridoxal 5′-phosphate (PLP) binding affinity and a different coenzyme binding mode compared with normal AGT. To shed light on the structural elements responsible for these defects, we solved the crystal structure of the variant to a resolution of 2.9 Å. Although the overall conformation of the variant is similar to that of normal AGT, we noticed: (i) a displacement of the PLP-binding Lys209 and Val185, located on the *re* and *si* side of PLP, respectively, and (ii) slight conformational changes of other active site residues, in particular Trp108, the base stacking residue with the pyridine cofactor moiety. This active site perturbation results in a mispositioning of the AGT-pyridoxamine 5′-phosphate (PMP) complex and of the external aldimine, as predicted by molecular modeling studies. Taken together, both predicted and observed movements caused by the S187F mutation are consistent with the following functional properties of the variant: (i) a 300- to 500-fold decrease in both the rate constant of L-alanine half-transamination and the *k*_cat_ of the overall transamination, (ii) a different PMP binding mode and affinity, and (iii) a different microenvironment of the external aldimine. Proposals for the treatment of patients bearing S187F mutation are discussed on the basis of these results. Proteins 2013; 81:1457–1465. © 2013 Wiley Periodicals, Inc.

## INTRODUCTION

Human alanine:glyoxylate aminotransferase (AGT) is a Fold-type I pyridoxal 5′-phosphate (PLP)-dependent enzyme, which catalyses the conversion of L-alanine and glyoxylate to pyruvate and glycine in liver peroxisomes.^1^ The protein is a 86 kDa-homodimer and each subunit comprises a 21-residues long N-terminal extension that wraps over the surface of the neighboring subunit and is important for the attainment of the correct conformation of the holo-protein,^2^ a 260-residues large domain containing most of the active site and of the dimerization interface, and a 110-residues C-terminal domain containing regions involved in the recognition and binding to the Pex5p peroxisomal receptor.^3^, ^4^ The AGT aminotransferase reaction follows a ping-pong bi–bi mechanism that relies on the formation of the external aldimine intermediate, in which the amino group of L-alanine replaces the ɛ-amino group of Lys209, the PLP-binding residue. Subsequent proton abstraction from Cα yields the quinonoid intermediate, and reprotonation at the C4′ of the coenzyme produces the ketimine intermediate. Hydrolysis of the ketimine intermediate yields pyruvate and the pyridoxamine-5′-phosphate (PMP) form of the enzyme. The second half-reaction in reverse direction with glyoxylate gives the enzyme in its initial PLP form and glycine.

Inherited mutations of the *AGXT* gene lead to the rare metabolic disorder Primary Hyperoxaluria Type I (PH1) (MIM #259900).^5^ In the absence of functional AGT, glyoxylate is converted to oxalate by cytosolic lactate dehydrogenase (LDH). Oxalate is an end-product of metabolism and is excreted in the urine. The increased production of oxalate in PH1 leads to the formation and deposition of calcium oxalate crystals in the urinary tract and kidneys causing urolithiasis and nephrocalcinosis. The subsequent renal failure results in the systemic deposition of calcium oxalate crystals.^6^, ^7^

More than 150 pathogenic mutations of the *AGXT* gene are associated with PH1 and the vast majority of them are missense point mutations that lead to the synthesis of an aberrant gene product.^8^ The molecular mechanisms by which missense pathogenic mutations cause AGT deficiency include the loss of catalytic activity, the reduced stability, the aberrant folding, the aggregation propensity, and the mislocalization of the protein.^1^,^9^–;^22^ Some mutations co-segregate and functionally interact with the minor allele of the *AGXT* gene encoding a non-pathogenic polymorphic form, which differs from that encoded by the more frequent major allele by a P11L and a I340M amino acid substitutions. Considering this heterogeneity of PH1 in terms of genotype and enzymatic phenotype, a crucial point for the understanding of the disease pathogenesis is the elucidation of the defect of each disease-causing variant at a molecular level. A fundamental step in this direction is the definition of the structural perturbations induced by the amino acid substitution on the AGT molecule and of the consequent alterations of the enzyme structural and/or functional properties.

The C→T mutation at position 682 in the major allele of the *AGXT* gene, leading to the S187F amino acid substitution, has been initially found in a PH1 patient showing less than 1% AGT catalytic activity with respect to normal subjects, as well as the nearly complete loss of AGT immunoreactive protein in liver biopsy. The genetic analysis revealed that the patient was heterozygous for the mutation, with the allele bearing the C_682_→T mutation producing an mRNA of normal size and abundance, while the other allele did not produce detectable amounts of mRNA.^23^

Ser187 is a large-domain residue conserved in the mammalian AGTs and belongs to a random-coil region that is not part of the active site.^4^ Cell-free expression studies have revealed that the S187F variant has reduced dimer stability and increased sensitivity to proteasomal degradation and that PLP is able to increase the stability of the protein.^14^, ^15^ We recently showed that the S187F variant in the recombinant purified form displays a reduced thermal stability in the apo-form with respect to normal AGT and that the presence of bound PLP is able to relieve the protein instability.^18^ Nevertheless, we found that the Ser-to-Phe substitution at position 187 also affects the AGT functional properties. In fact, the purified recombinant S187F variant has a reduced catalytic efficiency, an affinity for PLP increased of at least 27-fold, and a different coenzyme binding mode with respect to normal AGT.^18^ This implies that the variant might be characterized by structural changes remote from the mutation site with effects on the topography of the active site.

In order to have a deeper understanding of the structural and functional effects of the S187F mutation on AGT, we have solved the X-ray crystal structure of the S187F variant to a resolution of 2.9 Å. In addition, the putative binding mode of PMP and of the L-alanine external aldimine has been determined by molecular modeling studies. By comparing the properties of S187F with those of the normal protein, we showed that the Ser187-to-Phe substitution in human AGT leads to (i) active site modifications mainly consisting in the mispositioning of the PLP-binding lysine, (ii) an altered binding mode of pyridoxamine 5′-phosphate (PMP) and L-alanine external aldimine, and (iii) a reduced rate of the L-alanine half-transamination that could account for the reduced rate of the overall transamination of the variant.

## MATERIALS AND METHODS

### Protein expression and purification

The S187F variant in its His-tagged form was expressed in *Escherichia coli* and purified with the procedure already described.^24^ The apo form of the variant was prepared as previously described.^24^ The protein concentration in the AGT samples was determined by absorbance spectroscopy using an extinction coefficient of 9.54 × 10^4^*M*^−1^cm^-1^at 280 nm.^9^

### Crystallization and X-ray structure determination

S187F was concentrated to 5 mg/ml. Crystals were obtained by mixing 1 μl protein with 1 μl reservoir solution, comprising 0.1*M* HEPES (pH 7.5), 0.1*M* LiSO_4_, 23 % [w/w] PEG4000 and submitting to hanging drop vapor diffusion at 20 °C.

X-ray data were collected at X13 at the DORIS III synchrotron radiation storage ring, Hamburg, Germany. Data were processed with XDS^25^ and scaled with SCALA.^26^ Five percent of the reflections were randomly selected for cross validation. Initial analysis of the data suggested an orthorhombic space group, as all three unit cell angles were close to 90°. However, our attempts to solve the structure were unsuccessful. Consequently, we used Pointless^27^ to determine the correct space group (P2_1_). The structure of the S187F variant was solved by molecular replacement with Phaser^28^ using normal AGT as starting model (PDB entry: 1H0C).^4^ REFMAC^29^ was used to refine the initial structural model, applying translation/libration/srew parameterization, and including restraints from the non-crystallographic symmetry between the AGT chains. Manual building and structure analysis were carried out in COOT.^30^ The structure quality was assessed with MolProbity.^31^ Programs of the CCP4 package^32^ were used for average B-factor calculation (Fig. S1), structure manipulation, analysis, and validation.

### Kinetic studies

The half-transamination reaction of holoS187F (15 μM) with L-alanine (5–500 mM) was performed in 100 mM potassium phosphate buffer, pH 7.4, at 25°C in a total volume of 250 μl. The decrease in the 413 nm absorbance signal was measured as a function of time and fitted to the following equation:

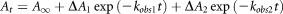
(1)
where *A_t_* is the absorbance at time *t,* Δ*A*_1_ is the amplitude of the fast phase, Δ*A*_2_ is the amplitude of the slow phase, *k*_obs1_ is the observed rate constant of the fast phase, *k*_obs2_ is the observed rate constant of the slow phase, and *A*_∞_ is the final absorbance.

The *k*_max_ and apparent *K*_m_^app^ values for each phase of the L-alanine half-reaction were determined by plotting the observed rate constants versus L-alanine concentrations and fitting the data to Eq. (2):

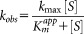
(2)

The coupling of the L-alanine half-transamination to LDH was carried out by performing the reaction in the presence of rabbit muscle LDH (0.5 mg/ml) and NADH (200 μM).

The detection and quantification of the PLP and PMP content during the L-alanine half-transamination was carried out using a previously described HPLC method.^9^

The kinetic parameters of the S187F mutant for the overall transamination of the L-alanine/glyoxylate pair in the absence and presence of 5 mM pyruvate were determined by measuring the amount of glyoxylate consumed by HPLC after derivatization with dinitrophenylhydrazine as previously described.^33^ Substrate saturation curves were fitted using Eq. (3), and dead-end inhibition data were fitted using Eq. (4) for competitive inhibition:

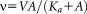
(3)

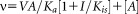
(4)
where *v* is the initial velocity, *V* is the maximum velocity, *A* is substrate concentration, *K*_a_ is the *K*_m_ for substrate A, and *K*_is_ is the slope constant.^34^

### Spectroscopic measurements

Absorption measurements were made with a Jasco V-550 spectrophotometer with a 1-cm path length quartz cuvette at a protein concentration of 15 μM.^35^ Visible circular dichroism spectra were recorded on a Jasco J-710 spectropolarimeter, by using 1-cm path length quartz cuvettes at an enzyme concentration of 10 μM. Routinely, three spectra were recorded at a scan speed of 50 nm min^−1^ with a bandwidth of 2 nm and averaged automatically. Both the instruments were equipped with a thermostatically controlled compartment at 25°C.

### Molecular modeling

The three-dimensional coordinates of the S187F mutant in the internal aldimine form were used as a starting point to generate the external aldimine and PMP bound forms of the enzyme, by means of energy minimization. These enzymatic forms of normal AGT and variant were generated using the BIOPOLYMER package from InsightII (V.2000, MSI, Los Angeles). PMP and external aldimine and were initially positioned into the active site of S187F, initially following the binding mode of PLP. Atomic potentials, partial and formal charges were defined using Cff91, a broadly used and already validated forcefield,^36^ whose analytical function and set of constants have been derived to best fit ab initio computed molecular orbital energy surfaces for a number of functional groups that are present in macromolecules (acids, alcohols, alkanes, alkenes, amides, amines, aromatics, esters, ethers, etc.). Validation of proper potentials, partial and formal charges was performed on PMP, internal and external aldimines, since after modifying the internal aldimine as previously described, the Cff91 potentials and charges were still unassigned. To this end, unrecognized atoms were manually identified and their potentials and charges fixed, after having manually verified: (i) type, based on Cff91 force field (this was done for polar hydrogen bonded to N of the pyridine ring, h*, and protonated nitrogen in 6-membered ring, nh+); (ii) hybridization (sp2 aromatic carbons); (iii) valences; (iv) formal charges; and (v) partial charges, re-computed with a semi-empirical method (PM3). Each complex was then subjected to further energy minimization. The minimization protocol adopted was based on a multi-step approach: first, all atoms except added hydrogens were fixed to allow hydrogens to adjust to the atomic environment. To this purpose, 1000 steepest descents steps were performed, a distance-dependent dielectric constant and a cut-off distance of 40 Å were used during each simulation, until the maximum energy derivative was less than 41.8 kJ·mol^−1^Å^−1^. Then main-chain atoms were fixed and side chains of every residue comprised in a sphere of 5 Å from the coenzyme were subjected to a gradually decreasing tethering force (from 4180 kJ·Å^−2^ to 418 kJ·Å^−2^) using conjugated gradients, until maximum derivative was less than 4.18 kJ·mol^−1^Å^−1^. Finally, a decreasing tethering force (until the system was totally relaxed) was applied on PMP and external aldimine, and every side-chain atom comprised in a sphere of 5 Å from PLP, using conjugated gradients until the maximum derivative was less than 0.0004 kJ·mol^−1^Å^−1^. Discover 2.9 and Analysis package of InsightII were used for minimization.

### Data analysis

The kinetic experiments were performed at least in duplicate, and in each case, the SEM was less than 10%. All data analysis was performed by non-linear curve fitting using Origin® 7.03 (OriginLab), and the errors indicated result from fitting to the appropriate equations.

## RESULTS AND DISCUSSION

### The S187F mutation affects the enzyme active site topology

**Figure 1:**
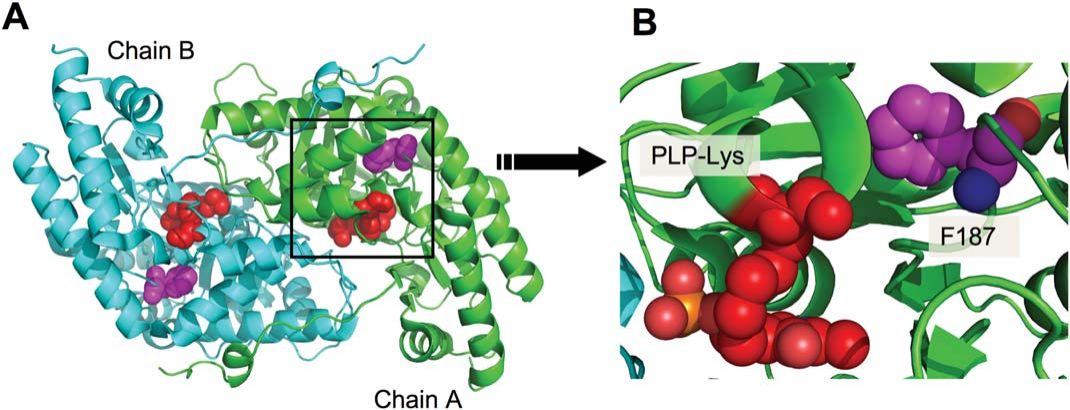
(a) Cartoon representation of the S187F variant dimer. The mutated amino acid (magenta) and the PLP-binding lysine (red) is represented by spheres. (b) Magnification of the area around the PLP-binding lysine. Because of the position of the phenylalanine mutation relative to the co-factor binding site, a conformational change is induced in the close proximity of the PLP-binding lysine. [Color figure can be viewed in the online issue, which is available at wileyonlinelibrary.com.]

To understand the molecular background of PH1 caused by the single amino acid mutation S187F, we have determined the crystal structure of the S187F variant at 2.9 Å resolution (PDB entry 4I8A, Table 1) (Fig. 1., Fig. S2). The asymmetric unit contains two AGT dimers, of which the complete polypeptide chain is visible in the final electron density, except for the N-terminal residues 1–5 (molecules A and C) or 1–4 (molecules B and D), and the C-terminal residues 391–392 of molecules B, C and D. Interestingly, the complete C-terminus, known as a peroxisomal targeting signal type 1 (PTS1), is visible in chain A, possibly due to the proximity of the N-terminal domain of molecule C that stabilizes the C-terminal loop (Fig. S3).

**Table I:** X-ray Structure Determination

	AGT(S187F)
**Data collection**	
Space group	P2_1_
Cell dimensions	
*a*, *b*, *c* (Å)	78.9, 101.6,116.8
α, β, γ (°)	90.0, 90.64, 90.0
Resolution (Å)	20.09–2.90 (3.06–2.90)
*R*_merge_	19.5 (47.6)
*I*/σ*I*	4.5 (1.9)
Completeness (%)	99.2 (99.9)
Redundancy	3.2 (3.2)
Wilson-plot B value	57.9
Measured reflections	130,322
Unique reflections	40,642
Unique reflections (*R*_free_)	2041
**Refinement**	
Resolution (Å)	18.07–2.90
Number of reflections	38 503
Molecules/ASU	
Protein	4
Glycerol	13
*R*_work_/*R*_free_	25.3/27.8
Number of TLS groups	20
Number of atoms	
Protein	11,851
Glycerol	78
*B* factors	
Protein	30.9
LYS-PLP	30.4
Glycerol	44.1
RMS deviations	
Bond lengths (Å)	0.011
Bond angles (°)	1.466
MolProbity validation	
Ramachandran favored (%)	98.4
Ramachandran outliers (%)	0.0
Rotamer outliers (%)	2.77

**Figure 2:**
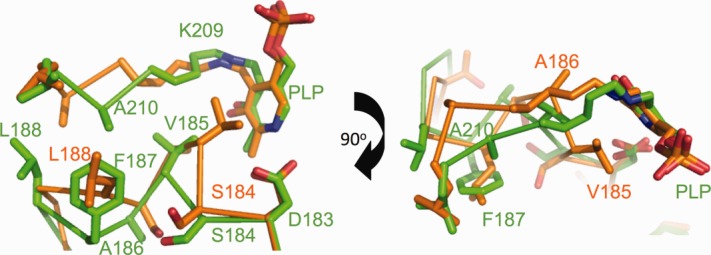
Superposition of the structure of the S187F variant (green) with that of normal AGT (orange) in the area of the cofactor binding site. Residues are labeled with the respective colors. The hydrophobic cleft that is formed by residues 185-188 triggers a conformational change in the loop where the PLP-binding lysine is located. [Color figure can be viewed in the online issue, which is available at wileyonlinelibrary.com.]

**Figure 3:**
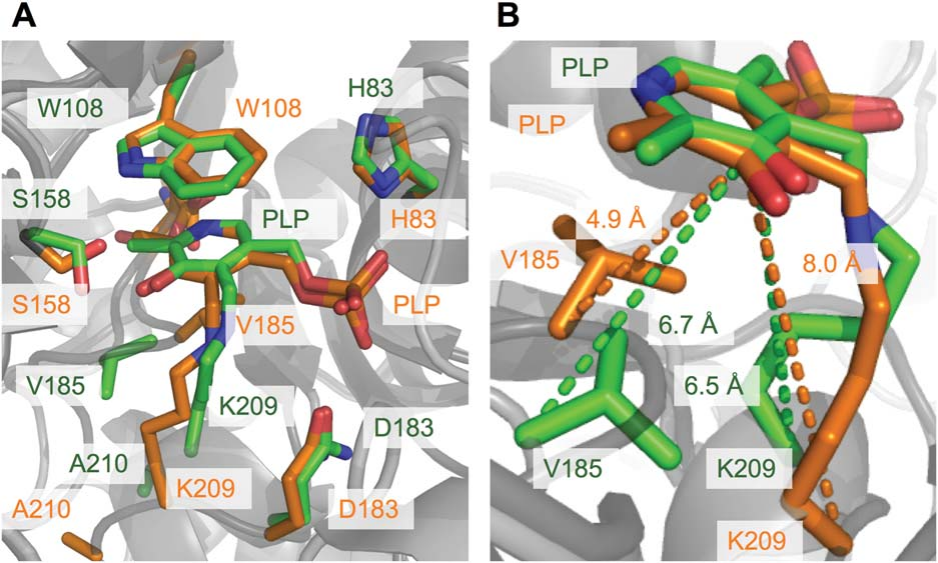
(a) Stick representation of the active site in the S187F variant (green) and normal AGT (orange). Because of the mutation, the main chain atoms of Lys209 in the mutant enzyme move closer to the PLP, which results in a shortened side chain conformation of Lys209. Other active site residues are only slightly affected by the mutation-induced structural changes. (b) Changes in the PLP-Lys209 and PLP-Val185 distances are represented by dashed lines in the respective colors. [Color figure can be viewed in the online issue, which is available at wileyonlinelibrary.com.]

The S187F structure superimposes well onto the normal AGT counterpart, with a root-mean-square deviation of 0.48 Å. Although the overall conformation of the two proteins is virtually identical, significant structural changes can be observed near the mutated amino acid (Fig. 2) as well as at the active site of the protein (Fig. 3). As a result of the mutation, the loop that contains Phe187 substantially changes its conformation, and moves Phe187 into the position of Leu188 of normal AGT. Because of this displacement, the Val185 residue of the mutant protein also changes position, and its side chain moves away from the PLP. The small hydrophobic pocket that is formed by Phe187 and Leu188 can accommodate Ala210, which eventually causes a shift of Ala210 and Lys209 towards Phe187 (Fig. 2). Although these conformational changes slightly alter the position of the PLP cofactor and of some active site residues (Trp108, Ser158, His83, and Asp183), they result in a significant displacement of Lys209 and the loop 183-188 (Fig. 2). In fact, the distance between the C_α_ of Lys209 and the C_3_ of the PLP methylpyridine ring decreases from 8.0 Å in the normal AGT enzyme to 6.5 Å in the mutant enzyme (Fig. 3), and the distance between the C_α_ of Val185 and the C_3_ of the PLP methylpyridine ring increases from 4.9 Å in the normal AGT enzyme to 6.7 Å in the mutant. Moreover, as calculated by Pocket Finder,^37^ in S187F the PLP cavity has a volume of 1073 Å^3^, while in normal AGT it has a volume of 949 Å^3^. These structural data allow us to explain how the mutation of a residue whose C_α_ is located at 10.5 Å from the C4′ of the coenzyme can affect the AGT functional properties so strongly. The shortened conformation of the lysine side chain observed could account for the reduced catalytic activity of the S187F variant, since Lys209 is a key residue taking part in the AGT catalytic mechanism, while the slightly altered position of several active site residues could account for the different PLP binding mode of S187F. Moreover, although it is difficult to understand the increased PLP binding affinity of the variant with respect to normal AGT,^18^ we speculate that it can be imputed to the increased volume of the PLP-binding pocket that could allow a better coenzyme accommodation.

### The S187F mutation affects the coenzyme binding mode and affinity as well as the external aldimine microenvironment

We investigated if the active site conformational changes of the S187F variant revealed by its crystal structure could affect the binding of other reaction intermediates besides the PLP coenzyme.^18^ We found that even a prolonged incubation time of apoS187F with PMP (up to 350 μM) does not result in the appearance of the positive dichroic signal at 340 nm, typical of the AGT-PMP complex formation.^9^ However, a direct measurement of bound PMP after 1 h incubation of the apo form of the mutant (10 μM) with 350 μM PMP in 100 mM potassium phosphate buffer, pH 7.4 (final volume of 500 μl) followed by concentration to about 10 μl by a Microcon device and four washing with 500 μl of the above buffer revealed that the retentate contains about 8 μM PMP. Thus, since only 40% of the variant is in the PMP bound form, the K_D(PMP)_ would be of ∼10 μM, at least 100-fold higher than that of normal AGT (data not shown).^9^ We have also monitored the spectral changes occurring in the S187F variant upon addition of D-alanine, an unproductive substrate analogue that mimics the formation of the external aldimine intermediate. The CD spectrum of S187F in the presence of a saturating concentration of D-alanine displays a positive band at 415 nm, 10-nm blue shifted with respect to that of normal AGT. Altogether, these data indicate that the variant is characterized by an altered PMP binding mode and affinity as well as by a different microenvironment of the external aldimine.

**Figure 4:**
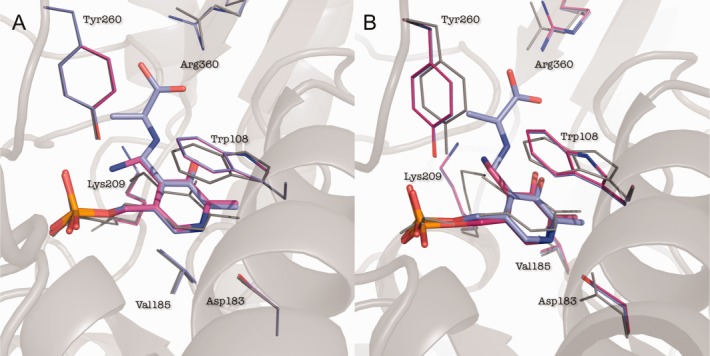
Modeling of the active site of the PMP- (pink) and L-alanine external aldimine- (cyan) bound forms of normal (a) and S187F (b) AGT. The internal aldimine (grey) is also shown for reference. Oxygen, nitrogen and phosphorous atoms are colored red, blue, and orange, respectively.

To have insights about the possible impact of the S187F mutation on the active site topography of these reaction intermediates, we compared docking models indicative of the putative binding mode of PMP and of the external aldimine with L-alanine [Fig. 4(a, b)] with those of normal AGT.^9^ The putative position of the manually docked molecules and their neighboring residues was relaxed by energy minimization means. Besides small changes related to the position of Tyr260, the most striking feature that distinguishes the S187F variant from normal AGT is the relative positioning of the coenzyme moiety and of Trp108. In fact, in S187F, both the AGT-PMP complex and the external aldimine are tilted by approximately 25° with respect to PLP towards Trp108 and Asp183 and the Trp108 side chain (on the *re* side of PLP) undergoes a remarkable conformational change, flipping by approximately 45° around the Cα–Cβ bond. The movement predicted is probably due to the need to accommodate the amino group of PMP or the substrate’s moiety and to avoid steric clashes with Lys209, and it is energetically impaired in normal AGT by the location of Val185 on the *si* side of the coenzyme.^9^ Although these represent simulated conformations, the predicted changes would result in loss of stabilization of PMP and the external aldimine on both *re* and *si* side of the pyridine ring. This is consistent with our experimental data and could account for the changes in both PMP binding mode and affinity and in the microenvironment of the external aldimine with D-alanine with respect to normal AGT.

### The S187F mutation decreases the catalytic efficiency of the L-alanine half-transamination

**Figure 5:**
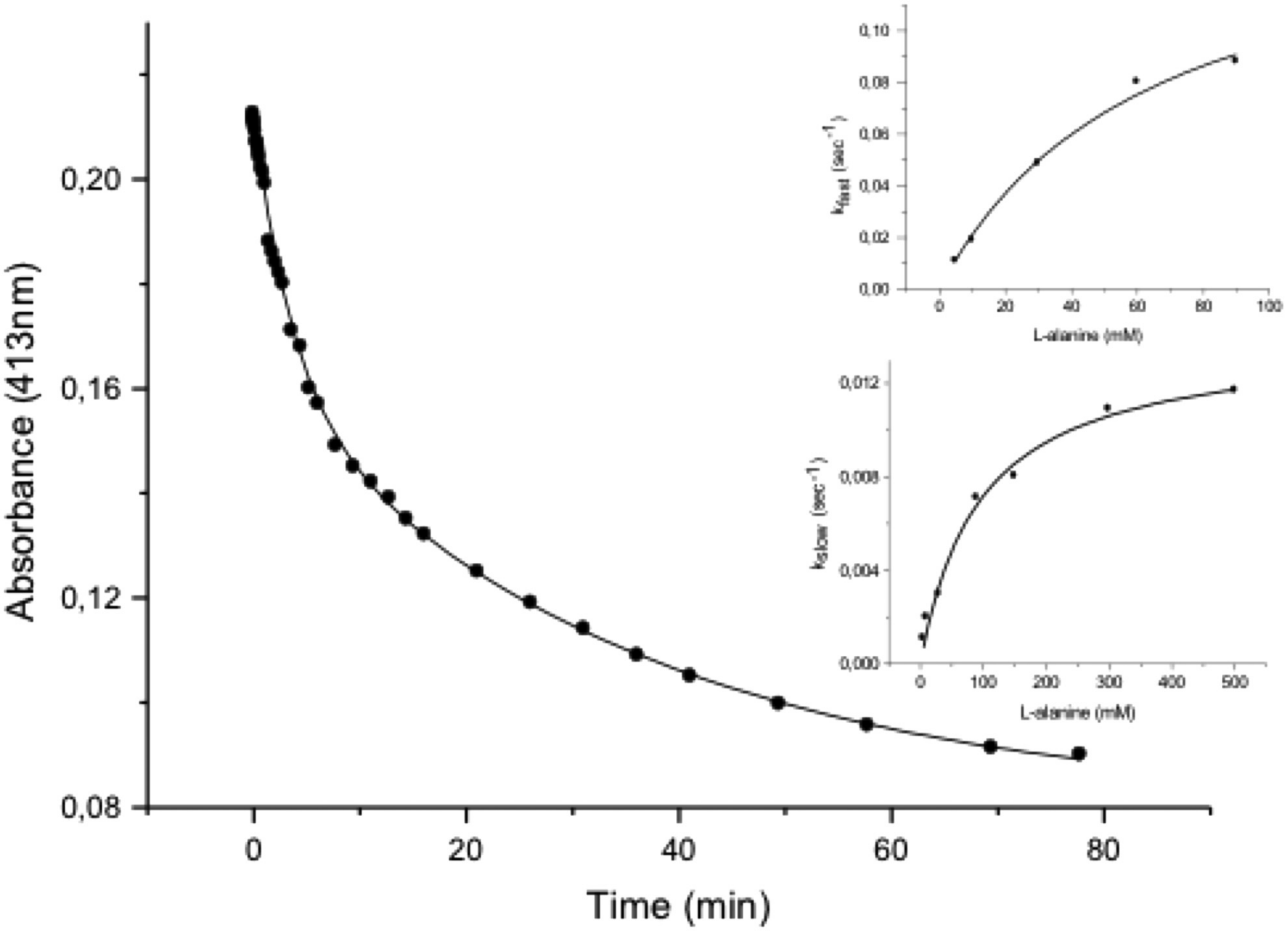
Time-dependent L-alanine half-transamination of the S187F variant. Plot of the 413 nm absorbance with time for the reaction of 15 μM S187F with 5 mM L-alanine. The line represents a two exponential fit. Insets: dependence of the rate constants (*k*_fast_ and *k*_slow_) as a function of L-alanine concentration. The lines represent the data fitted to Eq. (2).

We found that the S187F variant in the absence of exogenous coenzyme has values of *k*_cat_ for the alanine/glyoxylate pair, K_mL-alanine_ and K_mGlyoxylate_, equal to 0.147 ± 0.009 s^−1^, 52 ± 2 mM and 0.038 ± 0.008 mM, respectively. In order to investigate if the decrease in the catalytic efficiency was due to an impact of the mutation on the first and/or the second half-transamination reaction, we compared the kinetics of the L-alanine half-transamination of the variant (15 μM) with that of normal AGT. Similar to what is already observed in normal AGT,^9^ we could not detect the external aldimine with L-alanine and the quinonoid intermediates of the S187F variant. However, while both the decrease of the 429 nm absorbance band and the concomitant PLP → PMP conversion are single exponential processes in normal AGT,^9^ they display a biphasic behavior in the variant (Fig. 5). Both the apparent first-order rate constants, *k*_obs_, of the fast and the slow phases show a hyperbolic dependence on L-alanine concentration, with *k*_max_ and *K*_mapp_ values of 0.15 ± 0.02 s^−1^ and 64 ± 15 mM and of 0.015 ± 0.002 s^−1^ and 132 ± 38 mM for the fast and the slow phase, respectively. Thus, the S187F mutation strongly affects the first half-reaction by causing a ∼550-fold decrease of its catalytic efficiency with respect to normal AGT.^9^ This is not unexpected, considering the mispositioning of Lys209, as revealed by the crystal structure of the S187F mutant, and the role of the PLP-binding lysine in aminotransferases on C_α_ proton abstraction and C_4_′ reprotonation.^38^ According to this view, the replacement of a lysine in position 209 by an arginine residue completely abolishes the AGT catalytic activity.^39^ Since Lys209 is supposed to act as an acid/base catalyst in both half-reactions of AGT, an effect of the S187F mutation on the ketoacid half-transamination would also be expected. Unfortunately, this reaction cannot be monitored, because the apoenzyme of the S187F variant is unstable and prone to aggregation at the high enzyme concentration required to obtain an apo-PMP complex (at least 100 μM).

How can we explain the biphasic kinetics of the L-alanine half-transamination of the S187F mutant? The finding that the PLP binding kinetics is a monophasic process and that the kinetic constants of the L-alanine half-transamination do not change upon addition of D-alanine allow us to exclude the possibility that the S187F variant is found in solution as a mixture of a fast and a slow reacting form. Moreover, the fact that about 10 μM pyruvate is formed at the end of the fast phase the variant exhibits a *K_i_* value for the competitive inhibition of pyruvate against glyoxylate identical to that of normal AGT^9^ (2.2 ± 1.3 mM) suggests that the slow phase of the half-transamination is not due to pyruvate inhibition. Nevertheless, we found that (i) the L-alanine half-transamination becomes a single exponential process (with a *k*_obs_ value comparable to that of the fast phase) when it is coupled to the LDH/NADH reaction system, (ii) 100% of the PMP formed during the fast phase is bound at the AGT active site, and (iii) a 40% of bound PMP is present at the end of the slow phase. These results indicate that pyruvate is not released in solution in two kinetically distinguishable processes and that the slow phase is due to the accumulation of bound PMP that, in the presence of pyruvate, leads to an equilibrium between the forward and the reverse reaction. Consistent with this idea, a gradual recovery of the 413 nm absorbance is clearly detected at the end of the slow phase, indicative of the occurrence of the reversal transamination reaction.

## CONCLUSIONS

As already reported for other diseases involving PLP enzymes,^40^, ^41^ PH1 is characterized by a remarkable heterogeneity in terms of enzymatic phenotype. Thus, the best treatment strategy would depend on the specific molecular mechanism involved in the AGT mutation considered. Until now, the effect of many PH1-causing missense mutations has been rationalized in terms of the position of the mutated residue in the crystal structure of normal AGT.^4^ However, this approach does not always guarantee the elucidation of the molecular defect of each variant, as we show here with the S187F variant. The fact that the AGT catalytic activity and expression level in the liver biopsy of a patient bearing the S187F mutation was very low,^23^ along with the fact that the Ser187 residue is not part of the active site, could be mistaken as indications that this mutant only has a structural defect. In which case, treatment with pharmacological chaperones could potentially stabilize the folded conformation of the variant and would represent the best therapeutic strategy for patients bearing the S187F mutation. However, we had previously found that replacement of Ser187 by Phe in the recombinant protein caused both structural (reduced thermostability of the apo form) and functional (reduced catalytic efficiency and altered PLP binding mode and affinity) defects.^18^ Additionally, in the present work, we present the crystal structure of the variant paired with bioinformatic studies and show that the mutation causes a remarkable perturbation of the active site resulting in a mispositioning of (i) the PLP-binding lysine, (ii) the AGT–PMP complex, and (iii) the external aldimine. These data are consistent with biochemical analyses indicating a reduced rate of L-alanine half-transamination, a different PMP binding mode and affinity as well as an altered microenvironment of the external aldimine with D-alanine in comparison with normal AGT. The fact that even the folded variant exhibits structural defects at the active site responsible for a low transaminase activity, leads us to conclude that a therapy with pharmacological chaperones would probably only have limited effects for patients bearing the S187F mutation. The therapeutic strategies most likely to be effective in this case would be based on gene therapy or enzyme administration.

## Abbreviations

AGT: alanine:glyoxylate aminotransferase
CD: circular dichroism
*K*_D(PLP)_: equilibrium dissociation constant for PLP
*K*_D(PMP)_: equilibrium dissociation constant for PMP
PLP: pyridoxal 5′-phosphate
PH1: Primary Hyperoxaluria Type I
PMP: pyridoxamine 5′-phosphate.
