# Supplementary material for: Crystal structure of the S187F variant of human liver alanine: Aminotransferase associated with primary hyperoxaluria type I and its functional implications
Source: Proteins. 2013 Jun 1;81(8):1457–65. doi: 10.1002/prot.24300 (PMC3810726; doi:10.1002/prot.24300)
Supplement: Supplementary file 1 [file prot0081-1457-sd1.pdf]

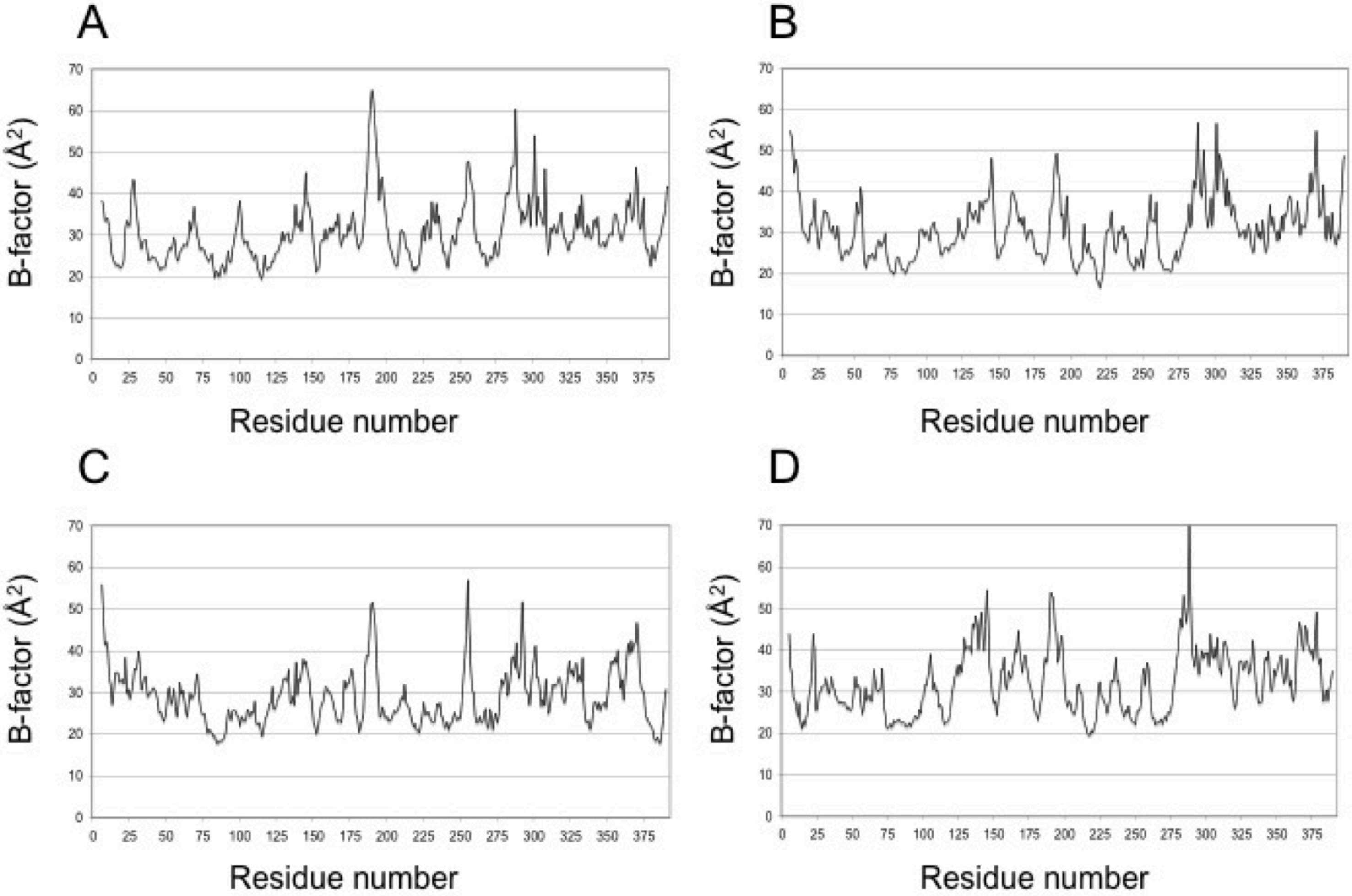

Figure S1. B-factor vs. residue plots of AGT(S187F) chain A (panel A), chain B (panel B), chain C (panel C) and chain D (panel D). The average B-factors of the residues were calculated by the software "Baverage" of the CCP4 package[31].
