# Supplementary material for: Crystal structure of the S187F variant of human liver alanine: Aminotransferase associated with primary hyperoxaluria type I and its functional implications
Source: Proteins. 2013 Jun 1;81(8):1457–65. doi: 10.1002/prot.24300 (PMC3810726; doi:10.1002/prot.24300)

Figure S2. Stereo image of the co-factor binding region of AGT(S187F).
The final electron density map is shown at 1 σ contour level.

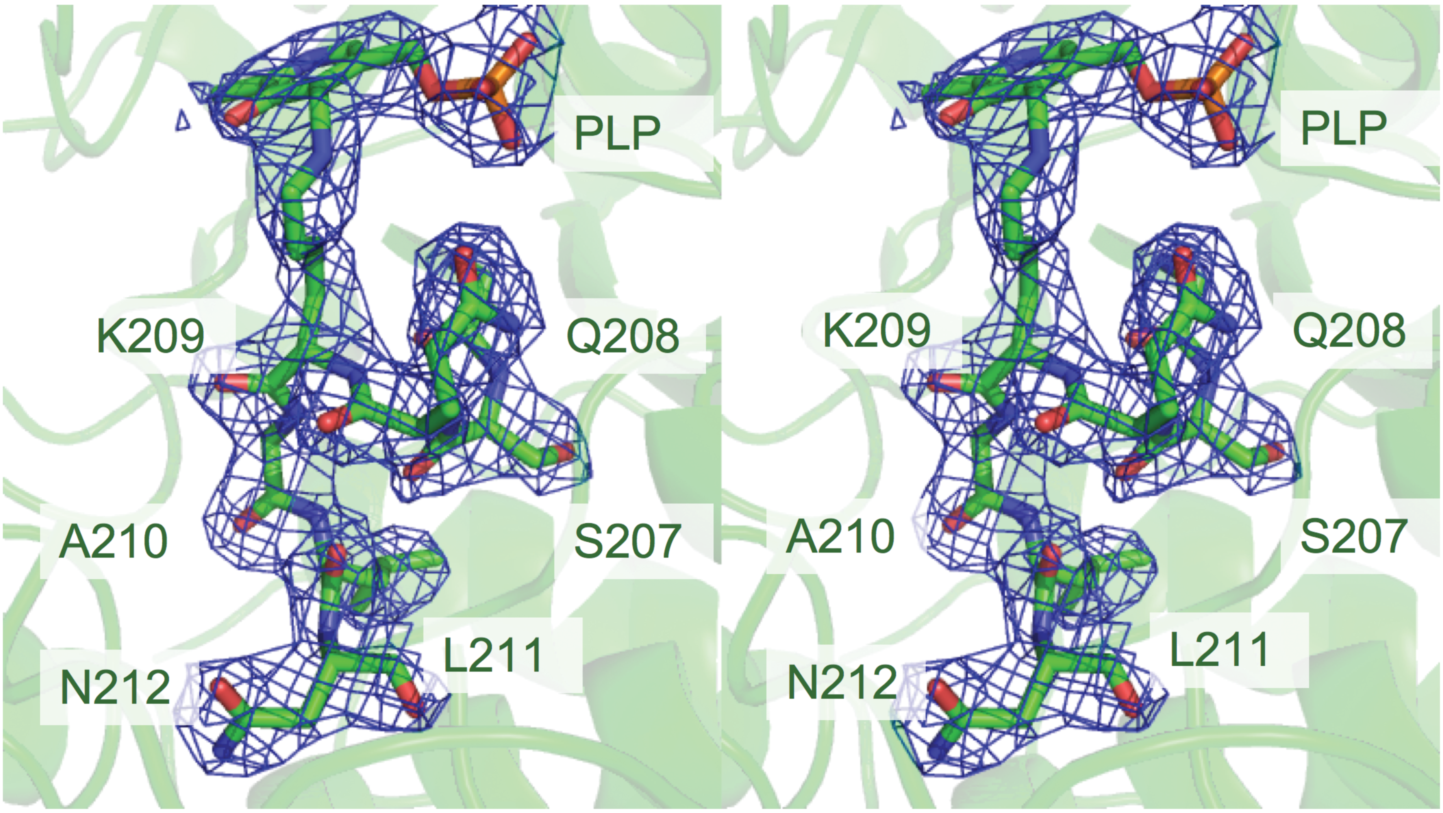

Supplement: Supplementary file 2 [file prot0081-1457-sd2.pdf]
