# Supplementary material for: Crystal structure of the S187F variant of human liver alanine: Aminotransferase associated with primary hyperoxaluria type I and its functional implications
Source: Proteins. 2013 Jun 1;81(8):1457–65. doi: 10.1002/prot.24300 (PMC3810726; doi:10.1002/prot.24300)

Figure S3. The peroxisomal targeting signal (PTS1) of AGT(S187F) in chain A, as it is visible in the electron density map at 1 σ contour level. The position of the flexible signal peptide is stabilized by chain C.

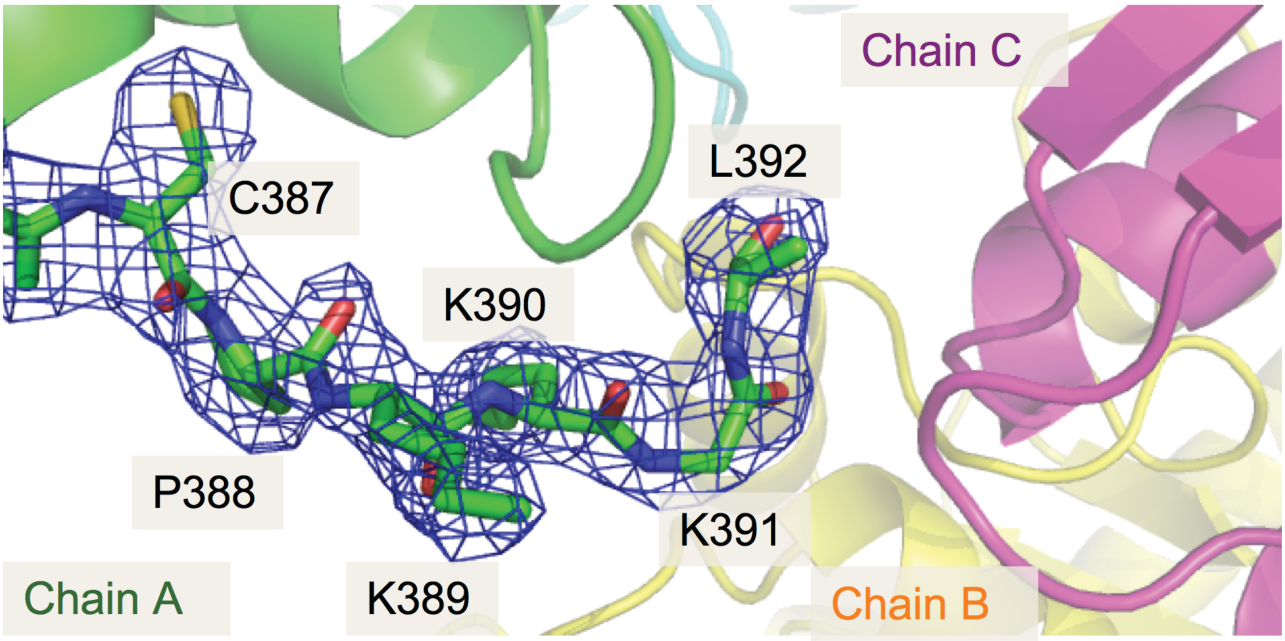

Supplement: Supplementary file 3 [file prot0081-1457-sd3.pdf]
